# First report of border disease virus in *Melophagus ovinus* (sheep ked) collected in Xinjiang, China

**DOI:** 10.1371/journal.pone.0221435

**Published:** 2019-08-20

**Authors:** Yong-Hong Liu, Bo He, Kai-Rui Li, Fei Li, Lu-Yao Zhang, Xian-Qiang Li, Li Zhao

**Affiliations:** 1 College of Veterinary Medicine, Inner Mongolia Agricultural University, Hohhot, People’s Republic of China; 2 College of Animal Science, Tarim University, Aral, People’s Republic of China; 3 Animal Loimia Controlling and Diagnostic Center of Aksu Region, Aksu, People’s Republic of China; University of Kansas Medical Center, UNITED STATES

## Abstract

*Melophagus ovinus* (sheep ked) is a blood-sucking ectoparasite that is parasitic primarily on sheep. It is widely distributed in different geographical regions worldwide. In China, it has been mainly found in Xinjiang, Gansu, and Tibet in recent years. In addition to causing direct damage to the animal hosts, *M*. *ovinus* also carries pathogens and serves as a vector for disease transmission. Border disease virus (BDV) is a positive-sense, single-stranded RNA pestivirus that mainly infects and causes border disease (BD) in sheep and goats worldwide. Since 2012, this disease has been reported in 4 provinces in China. In the present study, we investigated the presence of BDV in *M*. *ovinus* from Xinjiang and Gansu. Frozen *M*. *ovinus* collected during 2017 and 2018 from Xinjiang and Gansu and preserved in our laboratory were studied. First, total RNA of *M*. *ovinus* was extracted, followed by reverse transcription, PCR (RT-PCR) amplification of the 5′-UTR of BDV, and sequencing of the amplified products. Finally, the sequencing results were analyzed using DNAStar, MEGA 5.0 molecular biology software, and the BLAST online platform. The results from RT-PCR and sequencing analyses showed that among the samples included in the study, only the *M*. *ovinus* collected from Qinghe County in Alta, Xinjiang in 2018 tested positive for BDV. BLAST analysis showed that the viral strain with the most similar nucleotide identity to the sequence of the China/BDV/2018 fragment was the goat-derived BDV strain AH12-02 collected in Anhui, China, in 2012. A phylogenetic-tree analysis showed the strain to exhibit a BDV-3 genotype. This is the first report globally on BDV detected in *M*. *ovinus* and is also the first report of BDV discovered in Xinjiang, China. This study reconfirms the presence of BDV in China.

## Background

*Melophagus ovinus* (sheep ked) is a member of Hippoboscidae (Diptera: Hippoboscoidea) and is a blood-sucking ectoparasite of livestock and wild animals. *Melophagus ovinus* has a small head, strong mouthparts, no wings, dense bristles on the body surface, and 3 pairs of legs tipped with pointed claws [1–3]. The presence of *M*. *ovinus* has been reported in many countries in Africa, North America, Europe, Oceania, and Asia [2]. In China, *M*. *ovinus* has recently been reported mainly in Tibet [4], Xinjiang [2, 3, 5], and Gansu [6]. The direct and indirect damage caused by *M*. *ovinus* has led to huge economic losses in the sheep industry. In particular, *M*. *ovinus* can carry and transmit multiple pathogens and has thus become a worldwide concern [2, 3]. China has reported at least 13 pathogens detected in *M*. *ovinus* [2–6].

Border disease (BD) is a global disease caused by border disease virus (BDV) that mainly infects sheep and goats. BD is primarily characterized by vertical transmission causing congenital infections in fetus, reproductive disorders in ewes, persistent infections and transmission of pathogens in weak lambs [7, 8].

BDV belongs to the family *Flaviviridae* and the genus *Pestivirus*, and is a positive-sense, single-stranded RNA virus [9]. Pestivirus also includes classical swine fever virus (CSFV) and bovine viral diarrhea virus 1 and 2 (BVDV-1 and BVDV-2). The genome of *Pestivirus* is approximately 12.3 kb, with a single open reading frame between the 5′-untranslated regions (UTR) and 3′-UTR that encodes N-terminal autoprotease (N^pro^), capsid protein (C), 3 envelope proteins (E^rns^, E1 and E2), p7, and 6 non-structural proteins [10]. The 5′-UTR, N^pro^, and E2 genes are frequently used for genetic classification of novel virus isolates, and they provide consistent results [11–13]. As the 5′-UTR is relatively more conserved, it is used to define the pan-pestivirus reactive primer regions and is frequently used in genotyping studies [14]. Currently, there are at least 8 BDV genotypes from BDV-1 to BDV-8 [10, 15].

BD was first reported from the border region of England and Wales [7], and its presence is currently reported in Turkey, Japan, India, New Zealand, Australia, the United States, Canada, and many countries in Europe [7, 16]. Severe outbreaks of BD are uncommon [8, 17–19]; however, some studies have confirmed BDV epidemics in certain countries [8, 20–22]. In 2012, the isolation of BDV was first reported in China in the provinces of Anhui and Jiangsu from the serum and tissue samples of goats with persistent diarrhea [23]. In the same year, BDV was also isolated from serum samples of sheep in Jiangsu [13, 24]. The isolated BDV in both studies belonged to the BDV-3 genotype [24]. In 2012, the total positive rate of BDV antibodies detected in 5 regions of the Jiangsu province was 44.38%, of which the positive rate in that of sheep and goats was 33.33% and 46.32%, respectively [13]. In 2017, a report from China found that the positivity rate of BDV antibodies was 18.29% (400/2187) in the serum of *Ovis aries* from the Maqu County, Luqu County, and Tianzhu Tibetan Autonomous County in Gansu, as well as from Linzhi City in Tibet [25].

## Methods

### Study areas and *M*. *ovinus* collection

In June 2017, *M*. *ovinus* was collected from 5 sheep from a trading market in Yaha Town of Kuqa County in Aksu, Xinjiang (1029 m above sea level; 41°44′N, E83°14′E). Approximately 150 *M*. *ovinus* were collected from each sheep and were preserved at −70°C in our laboratory. Fifteen *M*. *ovinus* were randomly sampled from each sheep for use in the present study.

In March 2018, 12 *M*. *ovinus* were collected from 2 sheep from a peasant household in the Yumai Township of Aketedu County in Kizilsu Kirghiz Autonomous Prefecture, Xinjiang (1325 m above sea level; 39°13′N, 75°97′E) and were preserved at −80°C.

In March 2018, more than 400 *M*. *ovinus* were collected from 9 sheep from animal breeders in the Qinghe County in Alta, Xinjiang (1218 m above sea level; 46°67′N, 90°38′E) and were preserved at −70°C. Forty *M*. *ovinus* were randomly selected for use in the present study (Up to 5 sheep keds per sheep).

In June 2018, 130 *M*. *ovinus* were collected from 11 sheep from animal breeders in the Zhangyi Town of Liangzhou District in Wuwei, Gansu (2125 m above sea level; 37°56′N, 102°74′E) and were preserved at −70°C. Thirteen *M*. *ovinus* were randomly selected for use in the present study (Up to 2 sheep keds per sheep).

**Fig 1:**
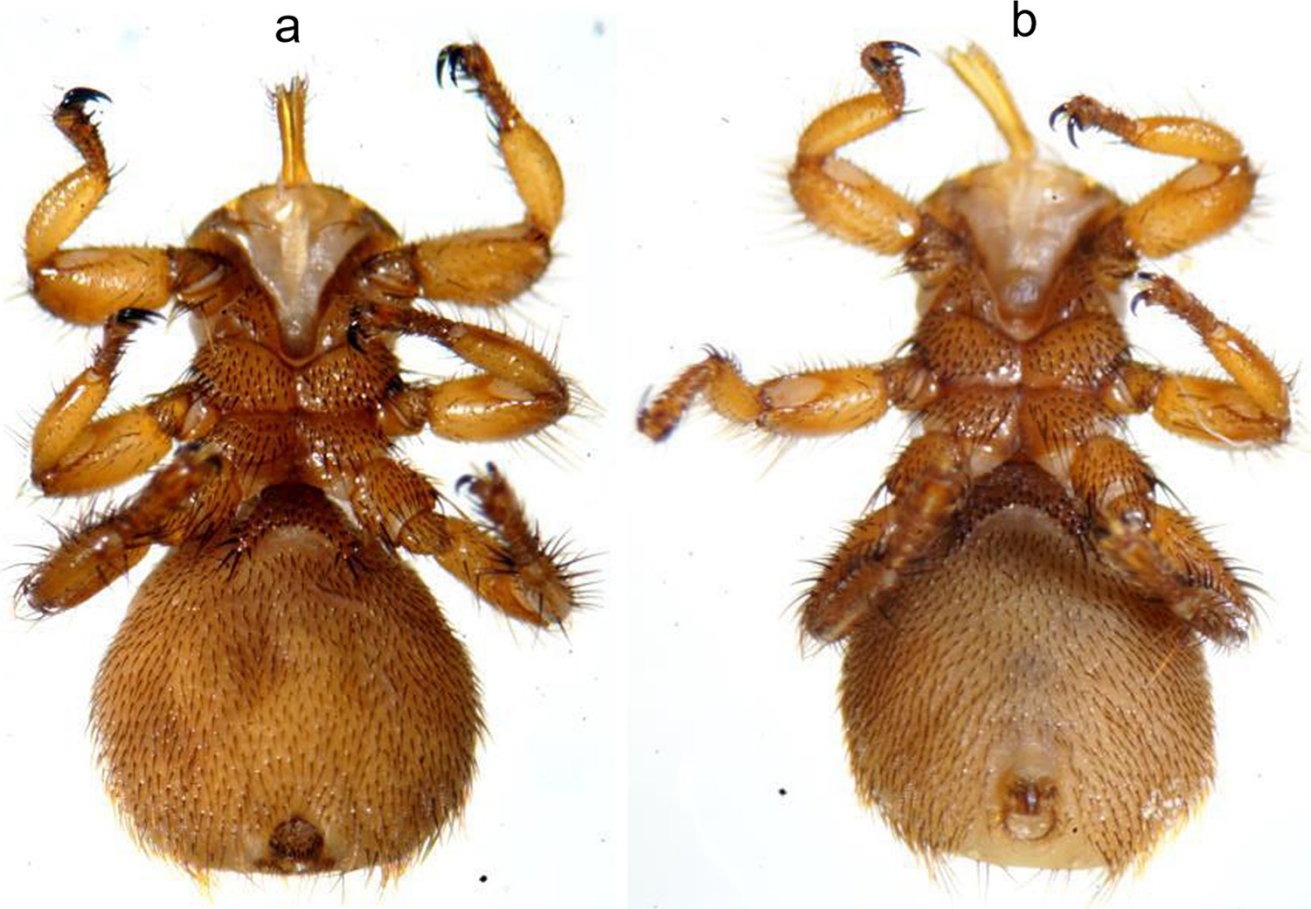
The ventral of Melophagus ovinus (a: Female; b: Male).

In this study, 140 (75 + 12 + 40 + 13) *M*. *ovinus* (Fig 1a and 1b) were processed individually.

### Ethics approval and consent to participate

Ethical treatment of animals was practiced in this study. Permission was obtained from the farm owners before collection of the specimens.

### Isolation of RNA, cDNA synthesis, PCR of the 5'-UTR, sequencing of PCR products and sequence analysis

The preserved and frozen *M*. *ovinus* were retrieved and placed in an autoclaved, chilled mortar. Liquid nitrogen was added and the samples were rapidly ground into powder. Next, total RNA from *M*. *ovinus* was extracted using the TaKaRa RNAiso Plus Kit (TaKaRa, Beijing, China, Code No. 9108) according to the manufacturer’s protocol. The precipitates were dissolved in 20 μL of RNase-free water in the final step. Next, cDNA was synthesized using the extracted RNA and according to the manufacturer’s protocol of the TaKaRa PrimeScript^™^ II 1st Strand cDNA Synthesis Kit (TaKaRa, Beijing, China, Code No. 6210A). Subsequently, the 5′-UTR of BDV was amplified according to the manufacturer’s protocol of Premix Taq^™^ (TaKaRa Taq^™^ Version 2.0) (TaKaRa, Beijing, China, Code No. R004A) and using the KOD-Plus amplification enzyme (Toyobo Co. Ltd, Osaka, Japan). The amplified product was approximately 225 bp.

Each 50 μl PCR reaction mixture contained 25 μl of the 2× PCR solution for Premix Taq^™^, 1 μl each of the forward and reverse primers (PBD1: 5'-TCGTGGTGAGATCCCTGAG-3'; PBD2: 5'-GCAGAGATTTTTTATACTAGCCTATRC-3' [21, 26]), 1 μl of the cDNA template, and distilled water.

The cycling conditions for the 5'-UTR amplification with primers PBD1 and PBD2 were as follows: initial denaturation at 94 °C for 5 min; 35 cycles at 94 °C for 30 s, 54 °C for 30 s, and 72 °C for 45 s; followed by final extension at 72 °C for 10 min.

The specific PCR amplification products were sequenced using an ABI PRISM^™^ 3730XL DNA Analyzer (ABI, Carlsbad, America). The sequences were aligned with reference sequences (Table 1) downloaded from GenBank using MEGA 5.0 software. The sequencing results were analyzed using the BLAST online platform (https://blast.ncbi.nlm.nih.gov/Blast.cgi?PROGRAM=blastn&PAGE_TYPE=BlastSearch&LINK_LOC=blasthome), as well as DNAStar and MEGA 5.0 molecular biology software, and were compared with the reference sequences (Table 1) downloaded from GenBank. The sequences were analyzed and a phylogenetic tree was constructed. The evolutionary history was inferred using the Neighbor-Joining method based on the Maximum Composite Likelihood method. The sequences obtained in this study were deposited in the GenBank database under the accession number MK322443.

**Table 1:** List of pestivirus strains used in this study.

GeneBank Accession No.	Strain	Year	Country	Host
AB122085	Casimir	2003	Germany	Wisent and reindeer
AF037405	X818	1987	Australia	Sheep lamb
AF144618	reindeer-1 V60-Krefeld	1996	Germany	*Rangifer tarandus*
AF220247	CP7-5A	1999	Germany	Bos
AJ829444	712/02	2004	Italy	*Capra hircus*
AM418427	BDV/Aydin/04-TR	2006	Turkey	Sheep
AM418428	BDV/Burdur/05-TR	2006	Turkey	Goat
AY453630	BM01	2003	Tunisia	Sheep
AY781152	/	2004	America	*Pronghorn antelope*
DQ361072	LE31C2	2001	Spain	Sheep
EF693988	89-F-5415	1989	France	Sheep
EF693989	90-F-6227	1990	France	Sheep
EF693991	90-F-6338	1990	France	Sheep
EF693993	91-F-7014	1991	France	Sheep
EF694003	06-F-0299/477	2006	France	Sheep
EU637006	chemnitz	1999	Germany	Sheep
FJ040215	Th/04_KhonKaen	2004	Thailand	Bovine
FM163379	LA/82/04	2010	Italy	*Ovies aries*
GQ902940	Gifhorn	2000	Germany	Pig
GU270877	H2121 (Chamois-1)	2002	Andorra	Chamois
HQ231763	Italy-1/10-1	2010	Italy	Cattle
HQ380231	CSFV-GZ-2009	2009	China	Pig
J04358	Alfort/Tuebingen	1989	Germany	Unknown
JQ946320	AH12-01	2012	China	Goat
JX437132	AH12-02	2012	China	Goat
JX437133	JSLS12-01	2012	China	Sheep
JX683184	JS12/04	2012	China	Goat
KF918753	Aveyron	1984	France	Sheep
KT072634	Italy-103761	2014	Italy	*Capra hircu*
KT327869	JSYZ15	2015	China	Sheep
KT327870	AHHX15	2015	China	Sheep
L49347	P97	1993	Taiwan	Pig
M96751	SD-1	1992	America	*Heifer*
NC_003678	giraffe-1 H138	1967	Kenya	*Giraffa camelopardalis*
NC_024018	/	2004	America	*Pronghorn antelope*
U18059	890	1994	America	*Heifer*
U65022	Moredun cp	1976	Scotland—Lothian	Sheep

## Results

The results from total RNA extraction of *M*. *ovinus*, cDNA synthesis, PCR amplification of the 5′-UTR of BDV, sequencing, and sequence analyses showed that only 7 *M*. *ovinus* from the Qinghe County in Xinjiang collected in 2018 were positive for BDV-specific PCR amplification. The sequencing results showed that the sequence of the 5′-UTR gene was identical in 7 samples, and the sequence was named China/BDV/2018.

In the GenBank database, the nucleotide identity between the China/BDV/2018 sequence and the goat-derived BDV strain AH12-02 isolated in Anhui, China in 2012, the pig-derived BDV strain Gifhorn isolated in Germany in 2000, the sheep-derived BDV strain 297 isolated in Slovakia in 2007, the goat-derived BDV strain AH12-01 isolated in Anhui, China in 2012, and the goat-derived BDV strain JS12/04 isolated in Jiangsu, China in 2012, were 94%, 93%, 93%, 93%, and 92%, respectively.

**Fig 2:**
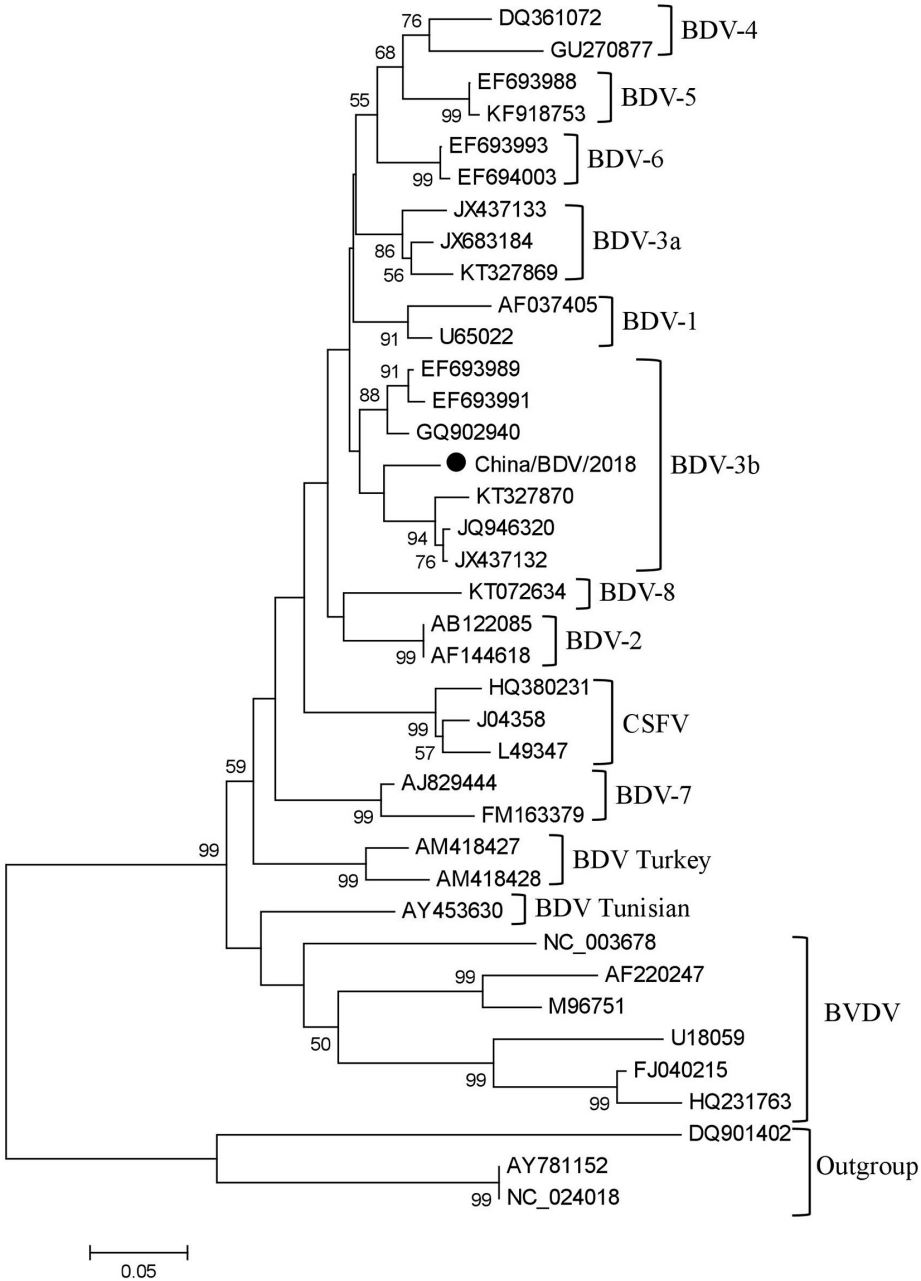
Phylogenetic tree of pestivirus based on 5'-UTR region. The evolutionary history was inferred by using the NJ method based on the Maximum Composite Likelihood method [27, 28]. The percentage of replicate trees in which the associated taxa clustered together in the bootstrap test (1000 replicates) are shown next to the branches [29]. The analysis involved 38 nucleotide sequences. There were a total of 191 positions in the final dataset. Evolutionary analyses were conducted in MEGA5 [30]. Sequences of this work were marked with black circular (●).

The 38 nucleotide sequences (Table 1) analyzed in this study included sequences from BDV-1 to BDV-8, BDV Turkey, BDV Tunisian, CSFV, BVDV-1, BVDV-2, and an outgroup. Based on the 191 positions in the 5′-UTR, MEGA 5.0 was used to perform the analysis on the evolutionary history of the strains. Viral strains from different countries, origins, and time periods could be clustered into 1 branch. China/BDV/2018 was classified as the BDV-3 genotype (Fig 2). However, the 10 BDV-3 genotype sequences were clearly divided into 2 smaller branches. Therefore, the subdivision of the BDV-3 genotype into BDV-3a and BDV-3b genotypes is recommended.

**Fig 3:**
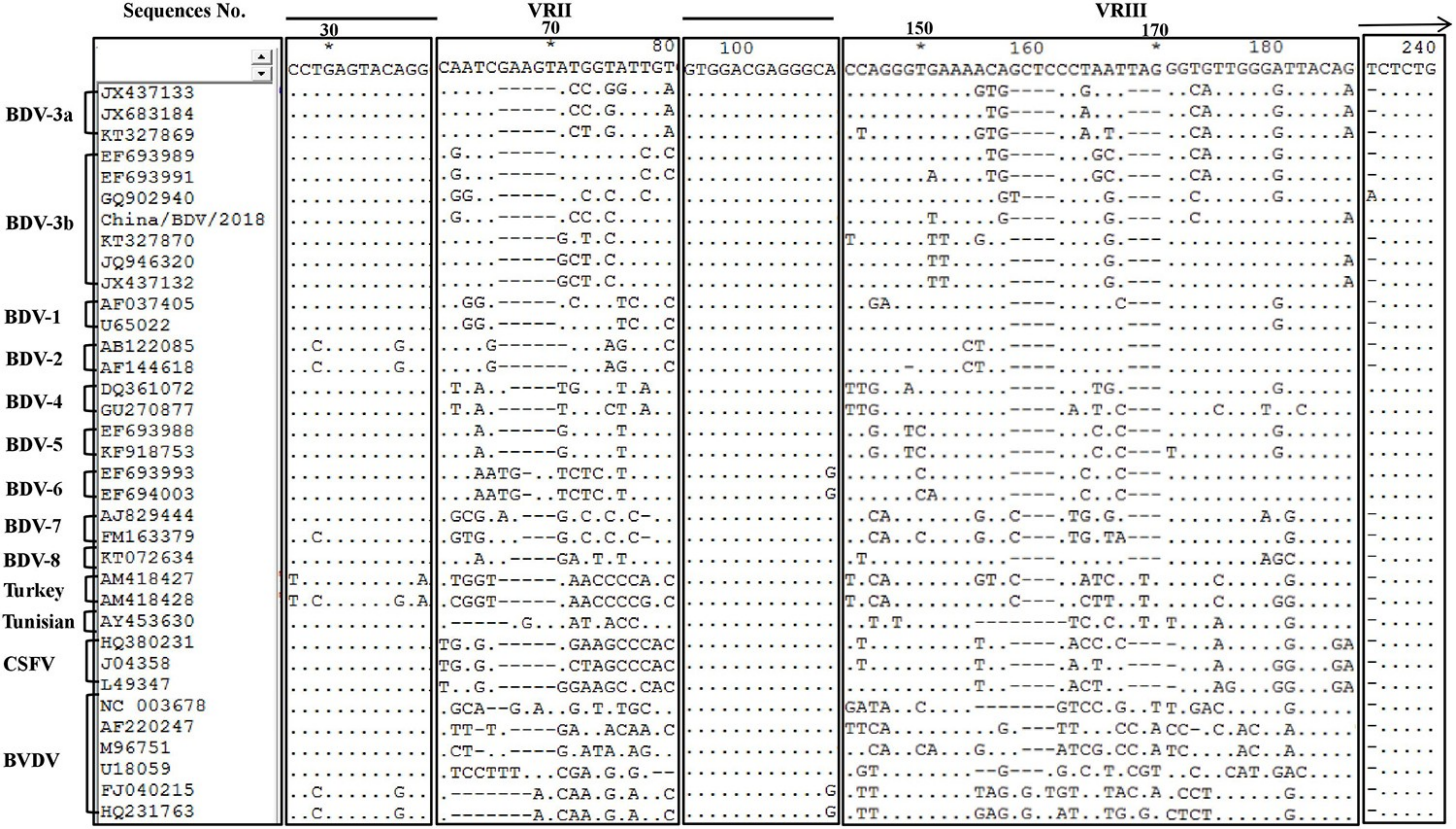
Alignment of nucleotide sequences from the 5'-UTR region of pestiviruses. Thick lines above the sequences indicate highly conserved regions in all pestiviruses studied. Two variable regions are double overlined (VR II and VR III, respectively). A pyrimidine-rich region is marked with an arrow.

Based on the analyses of the 5′-UTR of pestivirus, the nucleotide identity between the sequences of China/BDV/2018 and BVDV was 69.4% to 75.7%, between that of China/BDV/2018 and CSFV was 83.6% to 84.5%, between that of China/BDV/2018 and BDV-3 was 90.5% to 94.1%, and between that of China/BDV/2018 and other BDV subtypes was 78.8% to 89.6%. Analyses of the conserved and variable regions of the pestiviruses (VR II and VR III) showed that in the first conserved region, only BDV-2, BDV-7, and BDV Turkey had 1–4 base changes among that of all BDV strains, while in the second conserved region, only BDV-6 had a single base change among that of all BDV strains. The changes of BDV in VR II were prominent and the changes in VR III were minimal. Similar changes in the variable regions were observed in the same subtype of viral strains, including BDV-3b and BDV-3a. The viral strains of the BDV-3b subtype might be further classified into smaller divisions or exhibited greater variation (Fig 3).

## Discussion

Border disease virus (BDV) causes prenatal and postnatal infections in animals, resulting in reproductive disorders, birth of unviable lambs, and persistent infections [14]. In addition, mortalities can range from 40% to 85% in certain populations during epidemics [8, 20–22] and outbreaks in some countries [31], leading to huge economic losses. Furthermore, pestiviruses possess a high degree of genetic variability and extensive interspecies transmissions can occur between domestic and wild animals [32, 33]. Moreover, genetic changes in viruses can lead to changes in virulence [34]. A comprehensive analysis of reports from China since 2012, and evidence from etiological, molecular biology, and serological studies has confirmed that the sheep and/or goats in the Chinese provinces of Anhui, Jiangsu, Gansu, and Tibet have been infected with BDV [13, 23–25]. In summary, monitoring and research on BDV in China is necessary.

In recent years, *Melophagus ovinus* has been frequently reported in Tibet [4], Xinjiang [2, 3, 5], and Gansu [6] in China. At least 13 pathogens have been reported in *M*. *ovinus* found in China [2–6]. In this study, *M*. *ovinus* was collected from the body surface of sheep from Xinjiang and Gansu. The results showed that BDV was present in the *M*. *ovinus* isolated from sheep from Northern Xinjiang, which allowed us to make the connection that the sheep in Northern Xinjiang were infected with BDV. This confirms for the first time that *M*. *ovinus* is a carrier of BDV. This is also the first report confirming the presence of BDV in Xinjiang, China, which increases the number of BDV-positive provinces in China to 5.

Currently, the results of pestivirus genotyping using the 5′-UTR, N^pro^, or E2 sequences were identical and a consistent phylogeny [10, 14] was obtained. Among the 3 sequences, the 5′-UTR was the most conserved nucleotide sequence in pestiviruses. In addition, the N^pro^ or E2 genes lacked consensus sequences for primer design and sufficient reference fragments for analyses [14]. Therefore, the 5′-UTR is more frequently used in genotyping studies. This study also utilized the 5′-UTR to classify 38 nucleotide sequences, including the target sequence of this study. The results showed that China/BDV/2018 belonged to the BDV-3 genotype, which was consistent with the BDV classification in previous reports from China. BDV-3 is widely distributed worldwide, including in goats and sheep in Austria [35], Germany [33], India [36], Slovakia [37], Italy [38], Switzerland [39], China [12, 23, 24], and France [14], as well as cattle in Austria [35]. However, the BDV-3 genotype sequence analyzed in this study is clearly divided into 2 smaller branches. In addition, viral strains of the same subtype displayed similar changes in the variable regions, and these changes also suggest that BDV-3 can be further divided into 2 groups. This study suggests dividing the BDV-3 genotype into BDV-3a and BDV-3b genotypes, which will also reflect the greater diversity of BDV compared with other pestivirus species reported in the literature [13].

In this study, the nucleotide identity between the sequences of China/BDV/2018 and different subtypes of BDV ranged from 78.8% to 94.1%, while the nucleotide identity between the sequences of China/BDV/2018 and CSFV was 83.6% to 84.5%. This indicates that the nucleotide identity between the same type of pestiviruses may be much lower than that between pestiviruses and other types of viruses. In other words, the classification of pestiviruses based on nucleotide identity is unreliable, and the establishment of a phylogenetic tree is required. Furthermore, data from the GenBank database show that the sequences with similar identity with China/BDV/2018 are the goat-derived BDV strain AH12-02 isolated in Anhui, China in 2012, the pig-derived BDV strain Gifhorn isolated in Germany in 2000, and the sheep-derived BDV strain 297 isolated in Slovakia in 2007. These are BDV isolates from different regions, time periods, and origins. This information limits our ability to deduce the source and origin of China/BDV/2018. The emergence of BDV in Xinjiang may be related to animal trading, as there were no base changes in the 2 conserved regions in China/BDV/2018. The changes in the conserved region in all of the BDV strains listed in this study are relatively small, and this region may be used as a target site for primer design for BDV studies. Molecular epidemiological research and additional genetic studies on BDV should be extensively investigated in China and Xinjiang to provide definitive evidence for the classification, determination of origin, and control of BDV. Nevertheless, future analyses on additional BDV-3 isolates collected from different geographical regions in the world will help to provide a clearer picture in this regard.

## Conclusions

To our knowledge, this is the first report worldwide on the detection of border disease virus (BDV) in *Melophagus ovinus*. It is also the first report to confirm Xinjiang as the 5th BDV-positive province in China.

## Abbreviations

NJ: Neighbor-Joining
RT-PCR: reverse transcription-polymerase chain reaction
